# Valence and arousal of words in visual and conceptual interference control efficiency

**DOI:** 10.1371/journal.pone.0241694

**Published:** 2020-11-19

**Authors:** Kamil K. Imbir, Maciej Pastwa, Marta Jankowska, Marcin Kosman, Aleksandra Modzelewska, Adrianna Wielgopolan

**Affiliations:** 1 Faculty of Psychology, University of Warsaw, Warsaw, Poland; 2 Faculty of Polish Studies, Institute of Applied Polish Studies, Warsaw, Poland; University of Warsaw, POLAND

## Abstract

Cognitive control efficiency is susceptible to the emotional state of an individual. The aim of the current experiment was to search for the role of valence and arousal of emotion-laden words in a performance efficiency of a modified emotional Stroop task (EST) combined with the flanker task. Both paradigms allow for the measurement of the interference control, but interference appears on different stages of stimulus processing. In the flanker task, the interference is perceptual, while in EST, it is based on the emotional meaning of stimuli. We expected to find the effects of emotionality of words, that is, arousal and valence levels, for interference measured with EST. In a series of two experiments, the results confirmed that a high arousal level enlarges the reaction latencies to the EST. We also identified interaction between valence and arousal in shaping reaction latencies. We found the flanker congruency effect. We did not find interactions between emotional factors and flanker congruency. This suggests that interference measured with the EST and flanker task are in fact different from one another, and while using the modified EST combined with the flanker task, the word-meaning effects do not interfere with pure perceptual interferences.

## 1. Introduction

In everyday life we are surrounded by numerous, various stimuli, while our cognitive resources are limited. It is impossible to process all information at once [1], and from such a perspective, cognitive control plays an important role in human cognition [2]. It is a mental ability, referring to mechanisms and processes which allow us to reach our goals by selecting the relevant aspects of the situation, and ignoring those stimuli which are not important for us [3]. Limited mental resources with an excess of stimuli from the environment leads to conflict. This conflict can be resolved thanks to functions of cognitive control, such as working memory and response inhibition [4].

Cognitive control is in fact a complex mechanism [5]. Among basic subcomponents of cognitive control, updating (which can be explained as actualization and changing in working memory contents), shifting (ability to change task-set or goals) and inhibition (which refers to control over habitual and reflexive or prepotent reactions) can be mentioned [5–7]. Apart from these processes, interference seems to be especially important when the role of emotions in cognitive control is discussed. Interference control can be defined as an ability to inhibit inaccurate reactions and cognitions. This phenomenon may relate to the suppression of a competitive stimulus, the presence of distractions that may slow down the initial response or the hampering of internal stimuli that can interfere with ongoing working memory operations [4]. The interference itself is instead caused by automatic and involuntary processes that have to be stopped in order to execute the explicit task, due to the controlled and effortful processing. The interference control is therefore based on motor control (response inhibition) and cognitive inhibition in the field of working memory processes [5]. The interference control should also be susceptible to the properties, including emotional, of stimuli that are processed in order to gain the correct response [4], and thus paradigms designed to measure the interference control allow researchers to manipulate stimuli.

### 1.1. Cognitive control in Stroop and flanker tasks

Among tools for measuring the interference control component of cognitive control, the Stroop task [8] and the flanker task [9] are commonly used [3] to monitor the interference between the automatic and controlled processes in task solving. In the aforementioned tests, there are always at least two types of trials: congruent and incongruent. In the Stroop task, participants are asked to name the colour of the ink of presented word. In the congruent variant, the target (visual characteristic of stimuli—colour of the ink) and the semantic meaning of the presented word matches (“red” written in red ink). In the incongruent trial, the colour and the meaning of stimuli differ–(in this case, “red” written in green ink).

In the aforementioned Stroop task, two mechanisms occur. In congruent trials, participants can rely on automatic reading processes, which are well-trained and often used, which results in giving fast and accurate responses. Both the visual and semantic aspects of stimuli are convergent. The incongruent trial is more cognitively “demanding”–participants are asked to determine the visual aspect of stimuli, that is, the font colour of the word, even though they are automatically more prone to read the word and analyse its meaning. Completing this task requires interference control, which makes it possible to resolve the cognitive conflict. By inhibition of an automatic process, one can complete the task correctly [10]. Interference control helps in monitoring the conflict between task-relevant and task-irrelevant stimuli and reduces its impact on task performance [5].

The second tool mentioned above is flanker task [9]. In this task, there are analogically congruent and incongruent trials. Participants are asked to focus on the central target (e.g. arrow, word, number, letter), which is surrounded by the same or different stimuli. In the congruent trial, distractors indicate the same response as the target, conversely to the incongruent one. In some studies, there is also a “neutral” variant—when distractors are not assigned to the target [11].

As in the Stroop task, in the flanker task, the response is slower and less accurate in the incongruent trials [1,11–14]. In this trial, there is a visual conflict between target and distractors. Both flankers and targets are processed, so there is an interaction between two kinds of answer. In incongruent variants, the incorrect answer is primed, and it need to be suppressed. For this reason, the incongruent trial is more effortful and demands the interference control. That is why the reaction time is longer [11]. Spatial distribution of the incentives and flankers also affects interference [11].

The interference control is susceptible to the environmental stimuli properties and affective state of a person. The emotional state of the individual can influence the effectiveness of control processes over unwanted or distracting stimuli [15–17]. Emotions influence this process and play an important role in cognitive conflict resolution. Previous studies indicate that emotions affect attention processes: those which are positively valenced can broaden attention [18] and are associated with global and holistic processing [19]. Negative valenced emotional processes are said to narrow it [20] and lead to local processing [19].

In addition to the Stroop and flanker tasks, a good paradigm used to explore the impact of emotions on cognitive and attention processes is the emotional Stroop task (EST). Studies based on EST confirm that people are sensitive to the emotional characteristics of the stimulus, although it is not related to the task [21]. As in the classic Stroop paradigm, in EST, participants are asked to respond to the ink colour of various words. Some of these words have emotional meaning (e.g. slum or beauty), and some are neutral (e.g. chair). To complete the task correctly, the participant needs to ignore the semantic meaning of the stimuli [22]. Although the name suggests the similarity of both tasks, there are significant differences between the classical Stroop task and EST. The first is the lack of analogy to the processes of inhibition (in incongruent trials) and facilitation (in congruent ones) that occur in the classical paradigm. In EST, there is no semantic connection between the colour of ink and the meaning of the presented word. Interference occurs at a different level than in the classic Stroop task. There are no incongruent and congruent trials in EST. For instance, a neutral word–"chair"–printed in blue is no more or less congruent/incongruent than the emotional word "wicked" printed in red [22]. Incongruence results from the emotional load of the stimuli. In EST, incongruence is created precisely by the stimulus feature, which is emotional meaning. Research confirms that emotional traits of a stimulus are treated as a key factor and can affect attention and modulate the reaction time [21].

The combination of EST and flanker tasks proposed in the current study enables engaging two types of interference on two different levels. The flanker task provokes interference on perceptual level forcing an individual to ignore distractive competitive stimuli and select the target one. This type of interference requires suppression of distractors and result in higher response latencies for incongruent trials when individual needs to manage a tendency to spread attention between stimuli accompanying the target stimulus. The EST paradigm refers to higher processing oriented on word meaning detection. An individual is forced to ignore the emotional property of the stimuli and focus on the perceptual feature. This operation involves withholding automatic processes for effortful, voluntary processing. The consolidation of those paradigms was designed to incite both types of interference simultaneously. The colour flanker task on emotionally loaded stimuli was applied by Kanske and Kotz [23].

### 1.2. Emotional factors of valence and arousal in interference control

Two emotional concepts that are instrumental in understanding cognitive control are valence and arousal. Russell [24] stresses the significance of both valence and arousal, as he describes affect in terms of two separable dimensions. This gives us a chance to investigate the effects of valence and arousal in a separate way, which is particularly important given that there is enough evidence for their independent impact on emotions [25]. Therefore, we are able to influence the interference control needed to perform the task by manipulating one of the factors, valence or arousal [26–28]. Furthermore, valence and arousal refer to different phenomena: the former refers to pleasantness or unpleasantness of a given affective state; the latter is understood as the level of energy allocated to an affective state. Arousal is also traditionally understood as specific to the experiential system, which relies on emotional and simplified cognitive processing [29]. According to the core affect theory [30,31], valence and arousal are depicted as orthogonal dimensions: a person may experience a high level of arousal and at while reporting neutral feelings on the valence scale [32]. These two dimensions are fundamental to all emotions, that is, every emotion can be described in terms of valence and arousal.

Although valence and arousal are expected by the core affect theory [31] to be independent and orthogonal, studies focused on measuring those two dimensions showed a different relationship between these two affective dimensions, which takes the shape of a quadratic function [33–36]. Emotional stimuli located on the negative and positive end of the valence scale tend to be perceived as more arousing than stimuli which were of neutral valence. This pattern of relation between valence and arousal provides different conclusions than the one derived from Russell’s model [31], but the quadratic correlation seems to be more intuitive: highly emotional stimuli are often more arousing than neutral ones. As regards studies on interference control, one needs to be extremely cautious with the selection of stimulus material [37].

Valence and arousal were found to shape the interference control measured in EST and the flanker task. In EST, it usually takes less time to name the colour of font for a neutral word than an emotional one [21]. For instance, it was reported that in EST, subjects’ reaction times were longer for negatively valenced than neutral words [38]. In another study concerning EST, slower reaction times were observed when the stimuli in the task were emotional (both negative and positive in comparison to neutral) regardless of participants age: young or adult [39]. In a study involving a classic flanker task, it was observed that a positive mood resulted in slower reaction times to the target stimulus in comparison to neutral and negative moods [40]. In the study, it was also demonstrated that positive mood increased the processing of flanking distractors, thus increasing the breadth of attentional selection.

In the emotional version of the flanker task target and flanker stimuli differ, for example, in valence, and the task for participants is to discriminate emotional charge of a target stimulus on the valence scale. For example, when flankers were congruent (faces expressing the same type of affect as the target face), response latencies to the valence detection were faster in comparison to the incongruent trials [19]. This means that valence can cause the interference cost in the emotional flanker task. Such an effect was replicated with use of differently valenced stimuli [41,42]. For that reason, we may conclude that flanker interference may be also affected by emotional stimuli in a similar way as Stroop interference in EST. It was further shown that valence has an impact on interference control in three conflict tasks—flanker, Stroop, and Simon—showing that this impact occurs independently of the type of the used task [15]. In each of the tasks, participants were primed with a face stimulus (fearful, sad or neutral). It appeared that cognitive control effectiveness indexed by faster reaction times in incongruent trials was enhanced in the sadness condition in comparison to the fearful and neutral conditions. The impact of valence on cognitive control was also observed in another experiment involving a flanker task [43], The experimental design involved four mood conditions overall: two negative (anxious and sad) and two positive (happy and calm). The moods were induced by listening to excerpts of classical music and recalling a positive memory from the past. It was observed that negative valence of the emotional experience promoted reducing the interference caused by flankers. It was reported that only valence affected interference control and different arousal of the emotional states had no effect on conflict adaptation. Furthermore, it was observed that negatively valenced stimuli had a slowed down the reaction times in congruent flanker task trials, but not in the incongruent ones [44].

The effects of arousal were also found in the EST and flanker paradigm. It was observed that in an emotional Stroop task, highly arousing words caused emotional interference. This interference was present independently of valence [25]. Furthermore, an Eriksen flanker task was used to determine that moderate physiological arousal, induced by a set of physical exercises, facilitated processing of information in the congruent condition, but not in the incongruent condition [45]. The effect of arousal among neutral stimuli was also shown in a study using emotional words and flanker task [46]. It turned out that the shortest reaction times and most accurate responses were observed after moderately arousing stimuli in comparison to stimuli low and high on arousal scale.

On the other hand, there are studies reporting no interference of cognitive control caused by valence or arousal. For example, the performance of the participants taking part in a classic version of the Stroop and flanker tasks after listening to their favourite love songs (presented in order to induce an amorous mood) was influenced only by the intensity of their passionate love experienced, not by the valence or arousal of emotional stimuli [47]. In a classic study, Rowe, Hirsh, and Anderson [40], reported that positive mood enhanced cognitive broadening,. Another study however failed to replicate this result [48], regardless being divided into three experiments. In experiment 1 the positive mood was induced by recalling a positive memory and listening to classical music. In the flanker task used in the study, the stimuli were black letters N and H. Positive mood had no impact on cognitive control, and no effects reported by Rowe and colleagues [40] were found. This experiment was followed by experiment 2, where positive mood was induced by giving participants false positive feedback as regards their performance on the verbal interaction task. In experiment 3, the mood induction was carried out by asking participants to watch short film clips. The flanker tasks in both experiments were the same as in experiment 1. However, it was reported that no statistically significant effects of positive mood were found in any of the experiments [48]. Moreover, a set of experiments exploring effects of valence and arousal on cognitive control in a Stroop task turned out to be inconclusive [49]. Stroop performance in the reported experiment was the best in the high arousal group, and no effect of valence was found. It is worth mentioning that only two types of arousal (high or low) were distinguished, which may serve as an explanation as to why no consistent pattern of conclusions was discovered. The lack of unequivocal results might be due to distinguishing between an insufficient number of variables [47,49] or sample size [48]. Thus, in planning a study on cognitive control, one has to carefully develop the procedure so that the results will be reliable [37].

As we can see, the effects of valence and arousal may be observed in various types of tasks requiring cognitive control. The effects can be independent from one another [16,50–52] and can be observed in studies using classic [8,9] and emotional versions of both the Stroop and flanker tasks [19,25,38,53]. Research shows that in order to more accurately investigate the effects of valence and arousal, one needs to distinguish between three levels of these variables [46], as studies that investigated only two levels of valence or arousal turned out to be inconclusive [17,43,49].

### 1.3. Aim of the study and hypothesis

It is especially important in studies concerning the role of emotion in cognitive control to compare different types of interference control. The interference in the flanker task is rather perceptual (i.e. based on the perceptual features of the stimulus, like the direction or angle of dashes in the letters N and H [54], than connected with stimulus meaning, while in EST, the word stimulus’s emotional meaning itself causes the interference. For that reason, we have decided to combine the flanker task and EST in a single experimental procedure, comparing two stages of interference: (1) caused by the incongruence of stimuli colour (the same vs. different to the target stimulus); and (2) caused by the stimulus content (emotional vs. neutral). The main effects observed in the results of this study should be therefore associated with either perceptual interference (congruency) or meaning (emotional factors), while interactions of congruency with emotional factors should count for cross-exchange between flanker and EST interference controls.

We decided to prepare two different experiments, based on the aforementioned combination of EST and the flanker task. In the first experiment, we wanted to check whether interference caused by mere colour presented as the flanker distracter (emotion-laden words presented with the same or different colour in relation to the target, central word) would interact with EST-measured interference. In the second experiment, we decided to change the nature of flanker interference. It was still interference caused basically by the perceptual feature of an ink colour, but it was enhanced by the addition of meaning interference, that is, flanking words were colour-meaning words instead of emotion-laden words, always displayed in congruent way (i.e. word “red” displayed in red ink or word “blue” displayed in blue ink) in order to enhance interference. They still produced congruent flanker trials (the same colour for target and flanker stimuli) and incongruent trials (different colours for target and flanker).

The main aim of the following studies was to verify the role of valence and arousal in cognitive control. Since we decided on the modification of the EST paradigm as an experimental procedure, the expectations mostly concerned reaction latencies. We particularly expected the congruency effect for flanker interference: that is, incongruent trials should result in longer reaction latencies in comparison to congruent ones. We also wanted to check whether the “U”-shaped arousal effects observed in previous studies [46] can be replicated, when not only length of words and their frequency of usage are controlled, but also subjective significance of the word [55]. We assumed that there will be the main effect of arousal on the speed of performance, namely, the high and low arousing stimuli will cause the subjects to react more slowly than the moderately arousing ones. Furthermore, we expected interaction between emotional valence and arousal to be evoked by the words: emotionally valenced words (positive or negative) may speed up the reaction time in comparison to the neutral ones, especially in the highly arousing cluster of stimuli [56,57]. Finally, we wanted to verify the interactions between interferences caused by the flanker task and the emotionality of stimuli in EST, and we therefore expected interactions between the congruence of trials and the emotional properties of the words in response latencies.

## 2. Method

### 2.1. Participants

Two separate study samples, each consisting of fifty-nine participants (30 females and 29 males) voluntarily took part in the two experiments conducted at the University of Warsaw. In experiment 1, participants aged between 19 and 28 (*M* = 22.34; *SD* = 2.28) received a remuneration of 20 PLN (about 5 USD). Initially, the sample size was determined in advance as 60 participants (31 females and 29 males; *M* = 22.32; *SD* = 2.27), but the data from 1 individual were excluded from statistical analysis due to a large number of incorrect answers. In experiment 2, participants were between the ages of 19 and 32 (*M* = 22.34; *SD* = 2.54) and received a remuneration of 20 PLN (about 5 USD). Similarly to experiment 1, one subject from the initial group determined in advance as 60 participants has been excluded from analyses because of performing numerous incorrect trials.

Based on the studies using Emotional Stroop Task [36,54] and flanker task with emotional stimuli [43,45] we expected the *η*^*2*^ ranging from .1 to .21 for the significant effects regarding accuracy of the answers and from .15 to .35 for the effects regarding reaction times. We conducted a-priori power analyses for the study using G-Power 3 software [58]. The power analyses were conducted for the experimental design used in the whole set of experiments, where 3 factors are manipulated (3 valence levels x 3 arousal levels x 3 blocks of words), which gives 27 groups of stimuli, requiring 54 participants (with lowest possible *η*^*2*^ = .1) to achieve very high power of the study (.95). In the experiments reported in this study, we analysed the interaction of fewer levels of experimental factors (3 valence levels x 3 arousal levels x 2 congruence/incongruence of the stimuli), which means only 36 participants would be required to achieve the aforementioned power. For all the effects involving more measurements per factor (simple effects and interactions of two factors) the required sample sizes would be respectively smaller (ranging from 6 participants for the simple effects to 18 for the interactions of two factors). The reason why such small groups would be appropriate for identifying the effects in the study is its design, involving large numbers of repeated measures for each factor (e.g. for the simple effect of valence we compare 135 trials from each level of valence for each participant). We decided to use larger groups of participants than needed to identify possible effects of fewer sizes.

All participants were native speakers of the Polish language and had normal or corrected-to-normal eyesight. All were studying at the University of Warsaw at different faculties and colleges, such as linguistic studies, social sciences, engineering, humanities and art. The balanced proportion of the participation of students from various departments were kept.

We did not collect any personal data that would allow the participants to be identified and thus data were analyzed anonymously. The participants provided oral informed consent to participate in the experiment and this was documented in a research diary. The design, experimental conditions, and procedure were approved by the bioethical committee of the Faculty of Psychology at the University of Warsaw. All of the procedures involving human participants were conducted in accordance with the ethical standards of the institutional and/or national research committee, and with the 1964 Helsinki Declaration and its later amendments or comparable ethical standards.

### 2.2. Design

In both of the experiments, we were investigating the behavioural response to the trials of the flanker task based on the emotional verbal stimuli. The task for participants was similar to the EST (c.f. Procedure). The stimuli were matched for three levels of valence (negative, neutral and positive), as well as three levels of arousal (low, medium and high). The stimuli were also controlled for word length, their frequency of appearance in the Polish language and subjective significance. The display of the stimuli took place by two congruence conditions (congruent and incongruent) differing in appearance of flanker competition to the correct response.

The data were analysed using a repeated-measures analysis of variance (ANOVA) in a 3 (valence) x 3 (arousal) x 2 (congruence) design, with further pairwise analyses with the Bonferroni correction for significance levels. If the variables did not meet the sphericity assumption (measured by Mauchly’s test), the Greenhouse—Geisser correction was applied.

### 2.3. Linguistic materials

Four hundred and five words—nouns only—were used in the study, and were acquired from the Affective Norms for Polish Words Reload database [33]. To assess the values of the stimuli, in the study validating the database, fifty participants evaluated their affective response on eight different dimensions (arousal, subjective significance and valence were among them) for each of these words. The collected responses were calculated into means for every scale.

The experimental stimuli were divided into 9 conditions, diverging in the level of arousal (low, moderate and high) and valence (negative, neutral and positive). Each condition was composed of three different blocks of words, which gave 27 groups, 15 words each, groups presented in random order. Factors such as word length (the number of letters), frequency of their usage in Polish language [59] and their subjective significance were controlled. Subjective significance was initially treated as a factor, by which the stimuli were divided into three levels (low, medium and high), when preparing the word list. The list was prepared in such a way that every factor by which the stimuli were divided (valence, arousal and subjective significance) was a control factor for other dimensions, with equaled levels between groups (see S1 Appendix). In this particular experiment, we did not expect any effects regarding subjective significance, and therefore we treat it as a controlled factor, as the values on this dimension are averaged, when three stimuli blocks are put together as one cluster. As for the dimension of valence, mean ratings were *M* = 3.98, *SD* = 0.54 for negative, *M* = 5.12, *SD* = 0.22 for neutral and *M* = 6.15, *SD* = 0.46 for positive stimuli. In the case of arousal, stimuli of low arousal had mean ratings *M* = 3.34, *SD* = 0.26, moderately arousing words were *M* = 3.98, *SD* = 0.15 and highly arousing words were *M* = 4.75, *SD* = 0.41. All the words used in the experiment may be found in S1 Appendix.

To verify the stimuli selection, we conducted ANOVA analysis with a 3 (levels of valence) x 3 (levels of arousal) model. To actually validate the selection, we expected significant effects of valence levels on valence ratings (treated as a dependent variable in this case) and, similarly, effects of arousal on arousal ratings. No other effects should be significant, indicating that the groups are only different on the aforementioned dimensions.

We found significant differences between groups of valence divided by valence rating: *F*(2, 396) = 878.65, *p* < 0.001, η^2^ = 0.82. There was no effect for valence divided by arousal ratings–*F*(2, 396) = 1.60, *p* = 0.2, η^2^ = 0.008. There was also no significant differences between groups of valence on controlled dimensions, namely: *F*(2, 402) = 0.65, *p* = 0.52, η^2^ < 0.01 for the frequency of usage in Polish language (for this dimension, the data have been transformed into natural logarithms in order to achieve a results’ distribution closer to normal distribution), *F*(2, 402) = 1.09, *p* = 0.34, η^2^ < 0.01 for the number of letters and *F*(2, 402) = 0.49, *p* = 0.61, η^2^ < 0.01 for subjective significance.

As for the arousal, we found a significant effect for groups of arousal divided by arousal ratings: *F*(2, 396) = 775.90, *p* < 0.001, η^2^ = 0.80. However, we did not find any effect for arousal on the scale of valence–*F*(2, 396) = 0.69, *p* = 0.5, η^2^ = 0.003. There were no differences between groups of arousal on frequency of usage in the Polish language: *F*(2, 402) = 0.68, *p* = 0.5, η^2^ < 0.01; numbers of letters: *F*(2, 402) = 0.06, *p* = 0.98, η^2^ < 0.01; nor for the subjective significance: *F*(2, 402) = 0.025, *p* = 0.98, η^2^ < 0.01. S1 Appendix presents other analyses showing the validity of stimuli selection, as well as the mean values of assessments in manipulated and controlled variables for distinguished categories.

### 2.4. A combination of the flanker task and Stroop test

The task used in the current research constitutes a synthesis of the two most commonly used cognitive control tasks: the EST and the flanker task. A classical Stroop task demonstrates a cognitive interference effect thought the mismatch of presented stimuli, which reveals an incongruence between the visual feature of the stimulus and its meaning [8]. In the current study, the emotional version of the Stroop test was applied. In the emotional variant, subjects are asked to name the font colour of an emotion-laden word, and the effect of interference is based on a tendency to proceed emotionally significant stimuli with priority. Thus, it is harder to perform the explicit colour-naming task, which results in greater response latencies to the font colour of emotional words compared to neutral words [21].

The classical flanker task was developed by Eriksen and Eriksen [9], and it exemplifies the interference effect caused by the incongruence of visual attributes between the target and accompanying stimuli. The observed effect results from the similarity of spatial visual features, when two similar letters like “N” and “H” are used. An individual is asked to react to the central object while ignoring the accompanying letter (congruent or incongruent) located on the left and right side. The task may involve also using numbers, arrows or colour patches as a stimulus. The flanker can be located above and below the target object, or spatially. Based on the literature, it should be expected that incongruent trials will result in higher latency reaction time and greater errors in responses [1,12–14], as that task involves a perceptual interference control.

In both of our experiments, we used a combination of the flanker task and EST. The explicit task for participants was a detection of font colour of the emotionally charged word placed centrally on the screen. In experiment 1, two accompanying identical words (with the same meaning) were simultaneously displayed underneath and above the central object. The congruency referred to the font colour of the flankers—in congruent condition it was the same colour as for the target word, although in the incongruent condition, it was different. The procedure was based on a similar task proposed by Kanske and Kotz [57,60].

In experiment 2, the explicit task for participants was identical as in experiment 1. The only change was the flanker words, surrounding the emotionally loaded word. In this study, flankers were the names of the colours (red, orange, green and blue—accordingly, "czerwony", "pomarańczowy", "zielony" and "niebieski" in Polish). Flankers were always written in the corresponding colour fonts (e.g. "green" was always written in green font). Similarly to the experiment 1, congruency referred to the colour of fonts: in the congruent condition, the font colours of the word stimulus and flankers were the same, whereas in the incongruent condition, they were different.

**Fig 1:**
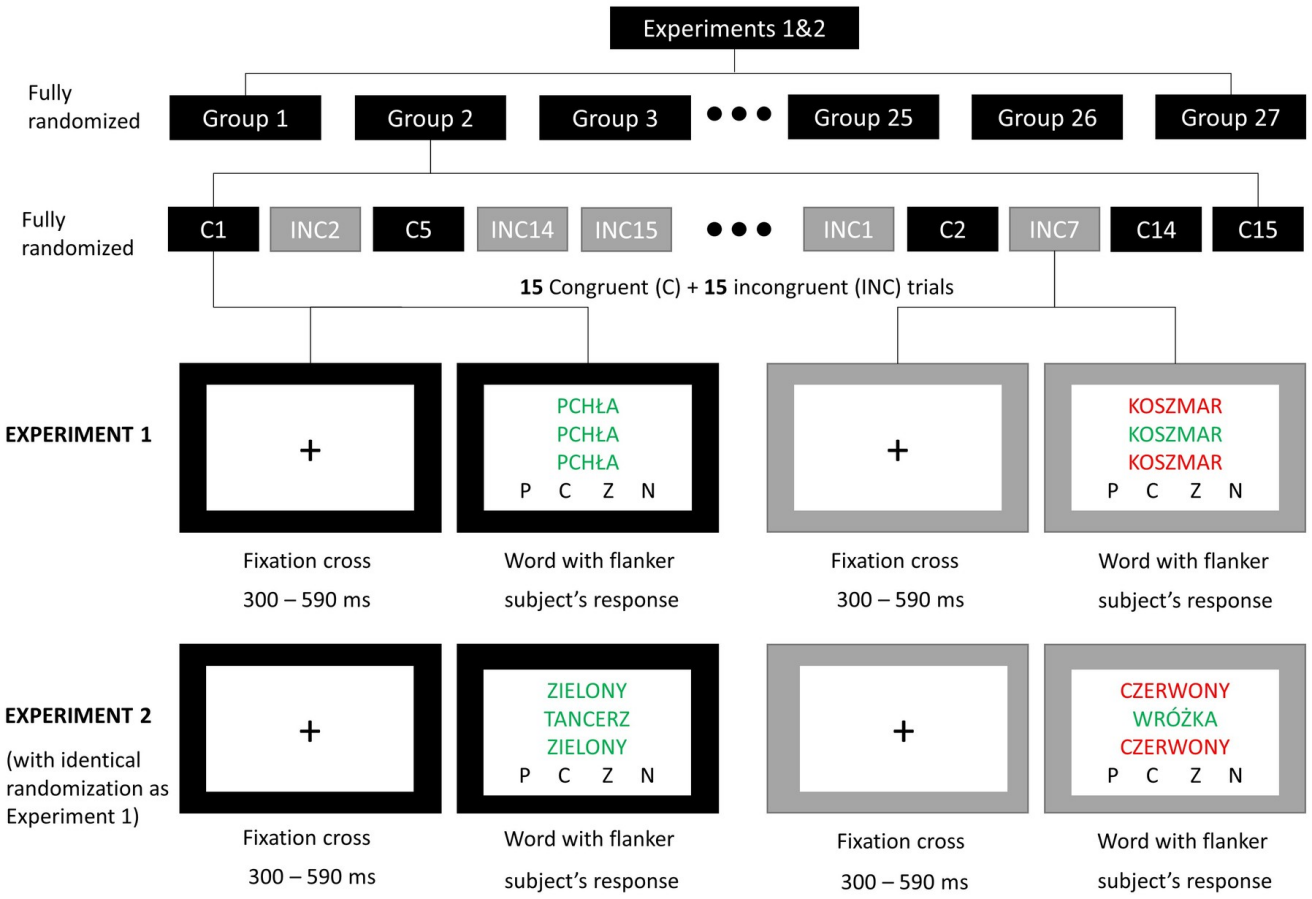
The outline of experimental procedures. Both experiments were built from 27 groups of words reflecting all factors manipulation. Groups were marked on the picture–“Group 1; Group 2; etc.”. Each group included both congruent–“C1; C2; C5” etc. and incongruent trials–“INC2; INC7; INC14” etc. One group consisted of 15 congruent and 15 incongruent trials. Participants were asked to name the font colour of the target word. The letters put on the picture represent colour names in Polish in the according order: P—orange (*pomarańczowy*), C—red (*czerwony*), Z—green (*zielony*), N—blue (*niebieski*). In the experiment 1, words used as examples are "pchła" (meaning "flea" in Polish) in the congruent condition and "koszmar" ("nightmare") in the incongruent condition. For experiment 2, the word example in congruent condition is "tancerz" ("dancer"), surrounded by flanker words "zielony" (meaning "green"). In the incongruent condition, there is the word "wróżka" ("fairy"), surrounded by flanker words "czerwony" (meaning "red").

Accordingly, the applied task provokes an interference control caused both by the necessity of processing an emotionally charged word and naming the font colour, as well as simultaneously ignoring the accompanying stimuli. Accordingly, an interference control was operationalized on two levels: a perceptual level and a meaning level. An additive impact of both interference effects should result in greater reaction times for incongruent trials. The trial of the task, described above, is presented in Fig 1, located later in the text.

### 2.5. Apparatus

The study was conducted with a standard laptop with a 15.6 inch LCD screen, and the distance was about 1m from the participant’s eyes. The font used in the procedure was Courier New 18-point size. The procedure was prepared with E-Prime 2.0 Software.

### 2.6. Procedure

Participants were told that the study is concerned with colour recognition. They were asked to name the font colour of central word and answer by pressing the correct key, marked on a keyboard. The first part of both of the experiments involved a training session, and during a sample task, participants could learn the correct performance. The training session was built from 18 single trials. Firstly, the fixation cross was displayed for a random time from 300 to 590 ms. Subsequently, the subject first named the font colour of the word in a classical Stroop task for four trials, and in the next stage, the subject named the colour of the square displayed centrally on the screen for four trials. Finally, the primary task used in the main part of experiment, a combination of the flanker task and the Stroop test, was applied, and ten trials were used. Participants pointed out the font colour of the target word using the letters indicating colour names and located below the target word. The following letters were displayed: P—indicates orange (*pomarańczowy*); C—indicates red (*czerwony*); Z—indicates green (*zielony*); and N—indicates blue (*niebieski*). Each participant was instructed to respond as quickly and correctly as possible.

Subsequently, during the main part of the experiment, participants were asked to name the font colour (red, green, blue or orange) of the emotionally loaded word. Before each trial, the fixation cross was displayed for a random time between 300 and 590 ms. The word was displayed at the centre of the screen, and it was surrounded by two additional words (flankers) with the same colour as the target word—presented in congruent condition, or with different colours—presented in incongruent condition. The detailed description of the main experimental task was included in the previous section (2.4. A combination of the flanker task and Stroop test). One word was displayed at 10% of the height of the screen below, and the other 10% above the main word, and all three words were aligned to the centre of the screen horizontally. There was no time limit for answering. Words were displayed in groups, and each group was built from 15 words displayed two times—once for congruent conditions and once for incongruent condition. Groups and words inside groups were randomized. The complete list included 405 words organized into 27 groups, reflecting all possible combinations of factor levels (3 valence x 3 arousal x 3 blocks of words (Blocks did not differ in levels of valence and arousal; however, they differed in subjective significance levels (low, medium and high))). The experimental procedures are illustrated in Fig 1.

## 3. Results

### 3.1. Experiment 1

There were 48,600 total trials (the number of trials was multiplied by the number of participants); 95.5% of them (46,422) were correct, and 4.5% (2178) of trials were deleted due to very long reaction times. Trials shorter than 300ms (43 trials; < 0.1%) were excluded from the analysis. The data from one subject were removed from the dataset due to very long reaction times. The final number of participants was N = 59.

The data were analysed with a 3 x 3 x 2 repeated-measures ANOVA. We performed two types of analysis: analysis for reaction times and analysis for correctness. Analyses were conducted using logarithm natural transformation of reaction times; transformation was conducted for the mean of each word; and p-values are reported with the Bonferroni correction. No significant effects were found for accuracy, and therefore this part of the analysis was omitted. Analyses of reaction times were done only on trials with correct answers.

We found a main effect of congruence (*F*(1, 57) = 18.22, *p* < 0.001, η^2^ = 0.24). We observed a significantly higher reaction time for incongruent trials (*M* = 937 ms, *SEM* = 24; natural logarithms (LN): *M* = 6.74, *SEM* = .02) compared to congruent trials (*M* = 916 ms, *SEM* = 23; LN: *M* = 6.72, *SEM* = .02; *t*(58) = 4.46, *p* < 0.001). There was also a main effect of arousal (*F*(1.76, 100.47) = 4.40, *p* < .05, η^2^ = 0.07). The data did not meet the sphericity assumption, and therefore, the degrees of freedom are reported with the Greenhouse—Geisser correction, ε = 0.88. Post-hoc tests showed that reaction time for moderate arousal (*M* = 916 ms, *SEM* = 22; LN: *M* = 6.73, *SEM* = .02) was lower than for high arousal (*M* = 937 ms, *SEM* = 26; LN: *M* = 6.74, *SEM* = .02; *t*(58) = -1.57, p < 0.05). We did not find the main effect of valence (*F*(2, 114) = 1.98, *p* = 0.14, η^2^ = 0.03), nor subjective significance (*F*(1.82, 103.49) = 0.89, *p* = 0.41, η^2^ = 0.02) to be statistically significant (the Greenhouse-Geisser correction = 0.91).

We observed a statistically significant interaction between arousal and valence *F*(4, 228) = 3.90, *p* < .01, η^2^ = 0.06. There was a significant difference in reaction times with regard to neutrally valenced words between words with moderate degrees of arousal (*M* = 909 ms, *SEM* = 22; LN: *M* = 6.72, *SEM* = .02) and highly arousing words (*M* = 970 ms, *SEM* = 29; LN: *M* = 6.77, *SEM* = .03; *t*(58) = -3.43, *p* < 0.01). Also, there was a significant difference between words with low levels of arousal (*M* = 923 ms, *SEM* = 24; LN: *M* = 6.73, *SEM* = .02) and words with high levels of arousal (*t*(58) = -2.79, *p* < 0.01).

**Fig 2:**
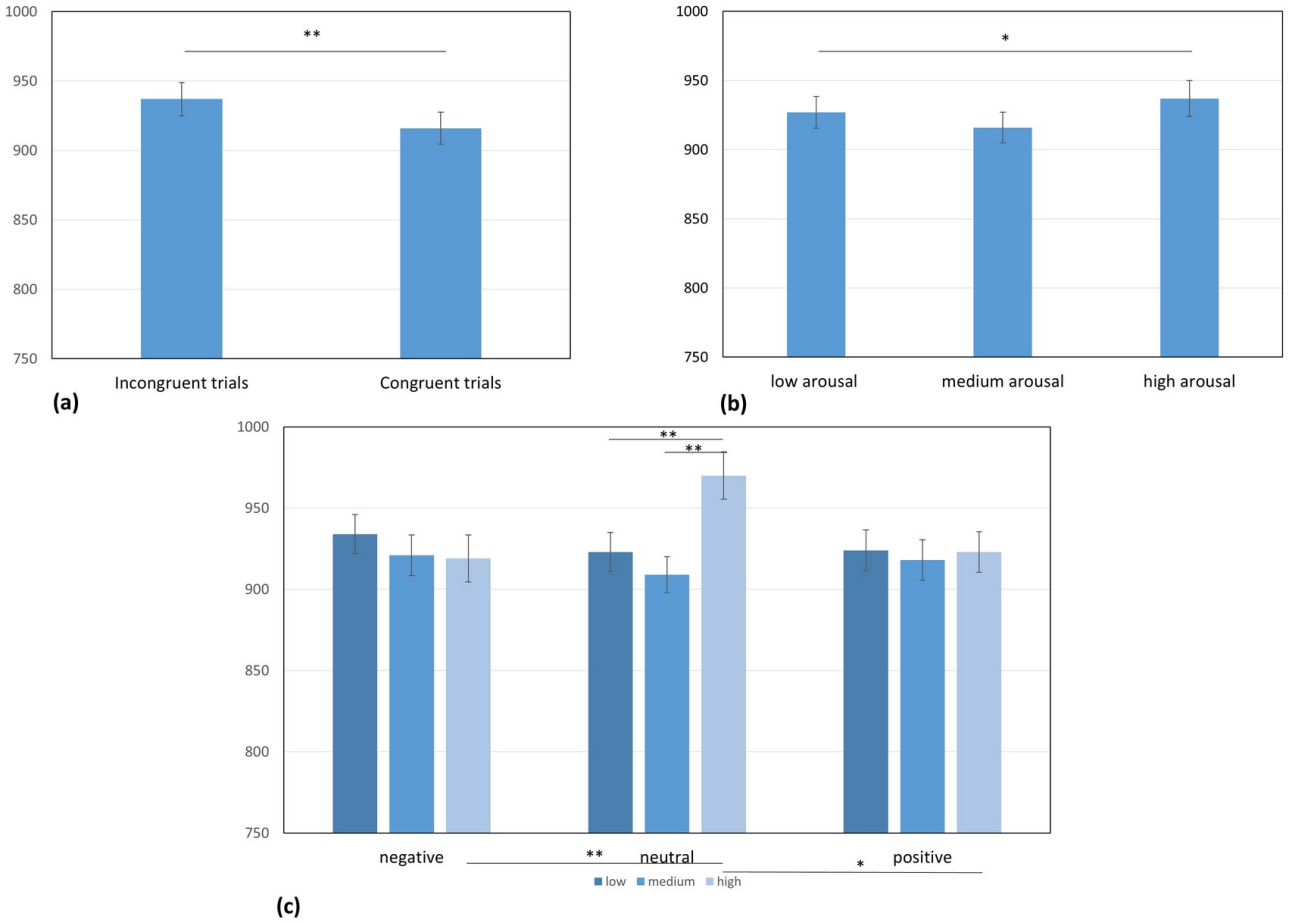
All of the statistically significant effects in experiment 1, illustrated with reaction times in milliseconds. On the top, there are main effects of (a) congruency for the incongruent and congruent trials, respectively (p < 0.01); and (b) arousal with statistically significant difference between medium and high arousal (p < 0.05). On the bottom, there is (c) the interaction effect of valence and arousal with significant difference in reaction times for neutrally valenced words in medium and high arousal variants and for low and high arousal variants. For highly arousing words, there is a significant difference between negative and neutral variants and for neutral and positive ones.

For words with high levels of arousal, there was a significant difference between negatively (*M* = 919 ms., *SEM* = 29; LN: *M* = 6.72, *SEM* = .03) and neutrally valenced stimuli (*M* = 970 ms, *SEM* = 29; LN: *M* = 6.77, *SEM* = .03; *t*(58) = 4.05, *p* < 0.01). We also found a significant difference between neutrally (*M* = 970 ms, *SEM* = 29) and positively valenced words (*M* = 923 ms, *SEM* = 25; LN: *M* = 6.73, *SEM* = .02; *t*(58) = 2,96, *p* < 0.04). We did not observe any other statistically significant interactions. The pattern for all of the differences is presented of Fig 2.

### 3.2. Experiment 2

In the second experiment, there were 48,600 trials (in total for 60 participants), with 93.8% of them being correct answers. Trials shorter than 300ms were deleted (862 trials– 1.8% of full database), as well as trials longer than 3 standard deviations calculated subject-wise (940 trials, 1.9%). Data from one participant were excluded from the analysis due to many mistakes. Therefore, the final number of participants was *N* = 59.

Data were analysed with a 3 (levels of valence) x 3 (levels of arousal) x 2 (levels of congruence) repeated-measures ANOVA with separate analyses for accuracy and time reactions. Time reactions were transformed using natural logarithms for the mean value of every word. All the *p*–values are reported with Bonferroni correction. If the data do not meet the sphericity assumption, the results are reported with the Greenhouse–Geisser correction. Analyses of reaction times were done only on trials with correct answers.

Accuracy was analysed using 3 (levels of valence) x 3 (levels of arousal) x 2 (levels of congruence) repeated-measures ANOVA. We found a main effect of congruence, *F*(1, 58) = 9.74; *p*<0.01, *η*^*2*^ = 0.144. In the congruent condition, there was a significantly higher percentage of accurate responses (*M* = 95%, *SEM* = 0.10) compared to the incongruent condition (*M* = 94.4%, *SEM* = 0.10); *t*(58) = 3.120; *p* < 0.01. No other significant differences regarding accuracy were found.

For reaction time analysis, we found a main effect of congruence *F*(1, 58) = 23.15; *p* < 0.001, *η*^*2*^ = 0.285. In the congruent condition, we observed shorter reaction times (*M* = 888ms, *SEM* = 23; LN: *M* = 6.70, *SEM* = 0.22) in comparison to the incongruent condition (*M* = 918ms, *SEM* = 21; LN: *M* = 6.73, *SEM* = 0.22); *t*(58) = 4.81, *p* < 0.001.

We also found a main effect of arousal *F*(1.771, 102.706) = 3.65; *p* < 0.05, *η*^*2*^ = 0.059. There was a significant difference in reaction times between the highly arousing condition (*M* = 913ms, *SEM* = 22; LN: *M* = 6.72, *SEM* = 0.22) and the low arousal condition (*M* = 895ms, *SEM* = 22; LN: *M* = 6.70, *SEM* = 0.23); *t*(58) = 2.95, *p* < 0.01.

**Fig 3:**
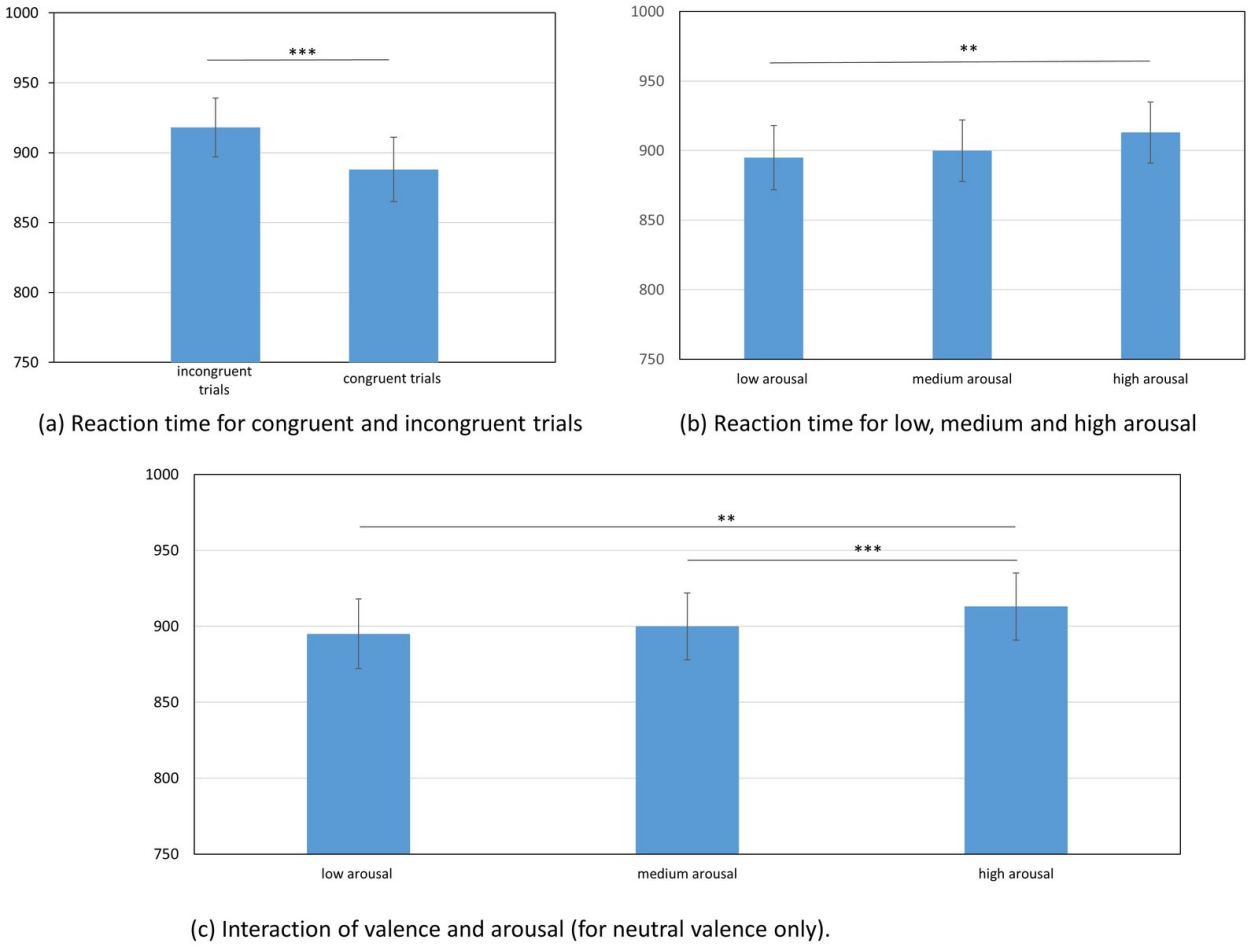
All of the statistically significant effects in experiment 2, illustrated with reaction times in milliseconds. On the top, there are main effects of: (a) congruency for the congruent and incongruent trials, respectively (p < 0.001); and (b) arousal, with a statistically significant difference between high and low arousal (p < 0.01). On the bottom, there is (c) the interaction effect of valence and arousal with significant differences between low and high arousal. as well as between medium and high arousal in the neutral valence condition.

There was an interaction effect between valence and arousal *F*(3.014, 174.837) = 3.122; p < 0.05; *η*^*2*^ = 0.051). For neutral valence, reaction times in the high arousal condition (*M* = 938ms, *SEM* = 26; LN: *M* = 6.75, *SEM* = 0.25) were significantly longer than reaction times in the moderate arousal condition (*M* = 898ms, *SEM* = 25; LN: *M* = 6.71, *SEM* = 0.25), *t*(58) = 4.09, *p* < 0.001. Timings in the high arousal condition (*M* = 938ms, *SEM* = 26; LN: *M* = 6.75, *SEM* = 0.25) were also significantly longer than in the low arousal condition (*M* = 884ms, *SEM* = 24; LN: *M* = 6.69; *SEM* = 0.24); *t*(58) = 3.45, *p* < 0.01. There were no other significant differences for this interaction. The pattern for all of the differences is presented in Fig 3.

## 4. Discussion

Analysing the results of both experiments, we can see that in general, they share the same pattern of effects. In regards to the congruence between colours of flankers and the target word, we found that incongruent trials elicited longer responses in both of the procedures. This effect has the same shape as differences in reaction times between congruent and incongruent trials in the Stroop task [1,11,12,14,47,61] and flanker task [1,40,47,48,62–64]. Observation of this effect confirms that the tasks employed for both of the experiments described in this study engaged cognitive control in a similar way, like the tasks that were the inspiration for developing these flanker-modified EST procedures.

In the second experiment, we observed the effect of congruence in the accuracy of responses, which did not play a role in the shape of the results of the first experiment. The incongruence of the stimuli in the first experiment played a role only on the visual level, namely, the colours of the target words and the flankers. The same kind of incongruence was involved in the second experiment, but it was also amplified by the content of the flanker, which was the name of the colour in which the flankers were written. We may conclude that this involves incongruence on a different, later level of processing the stimuli [14,65], namely, the moment of deciding about the response. When a subject verbalizes the response, which in this task (as in the EST) is the name of the colour, the names of the colours displayed on the screen may affect the process, disturbing the verbalization and changing the response. This may be the reason why the accuracy effect was observed only in the second of two experiments. The effect of accuracy observed in the second experiment is in fact similar to the Stroop effect, as naming the target colour was interfered with by reading the content of the flankers [12].

The arousal effects share the same shape among the two experiments, namely, the words with a high load of arousal elicited longer reactions than the mildly arousing ones, no matter if they were displayed three times, one under the other (Experiment 1), or if they were the only emotional word on the screen (Experiment 2). These results are congruent with the previous findings about the impact of arousal in processing tasks requiring cognitive control [17,21,25,26,39,46,51,52,66,67].

No main effect of valence was observed in the current experiments, which is in contrast to many of the published results [26,38,44,50,62,68]. This sheds even more light on the role of arousal, as some recent studies already reported the lack of pure valence effects in the procedures requiring cognitive control, especially when arousal is orthogonally crossed with valence [56,60,61,63,64].

It is also interesting that an interaction was found between arousal and valence of the word in both experiments. In the group of neutral stimuli, the responses in trials with highly arousing words were longer than in trials with mildly arousing words and the ones low on arousal. In particular, the higher the load of arousal in the group of neutral words, the longer the reaction time. This effect looks similar to the main effect of arousal, but we can conclude that stimuli low on arousal affect the cognitive control differently only once stripped out of its negative or positive connotations. This interaction effect is also similar to the effects of interaction between valence and arousal previously described in the literature [12] as a low level of arousal promotes faster reactions within certain valence clusters. Some researchers also reported interactions between valence and arousal, but the design of their experiments did not allow excessive control over both of those emotional dimensions [25,26].

In the first experiment, the interaction between the arousal and valence also manifested itself in the cluster of the highly arousing words, where the reaction times differed between the neutral ones and the emotionally valenced ones: processing the positive or negative words shortened the latencies. As mentioned earlier, the dimensions of valence and arousal are separate [31], and therefore they are correlated in a particular manner—usually the words with an extreme charge of valence (negative or positive) also have a high load of arousal, which is part of the natural mode of emotional processing. The words high on arousal but neutral on the dimension of valence may be a specific group, causing the largest slowdown in the reaction latencies. The task employed for the second procedure was more difficult, as it required cognitive control not only on the basically visual level (colour of the flankers), but also on the spelling/verbalization level (the content of the flankers). The level of focus required in the second experiment may be responsible for wiping out the effect in the group of highly arousing stimuli.

It is important to note that there was no interaction observed between the congruence of the trial and the emotional properties of the target. The emotional factors played their role in the length of delivering the response, but they did not affect the disturbance provided by the incongruence between the flankers and the target. This stands in contrast to an entire set of studies where emotional factors affect the effects of flankers [40,43,44,60–63]. In the modified EST tasks proposed in this study [57,60], emotional factors were operating in the exact same moment as the incongruence between the flankers and the target—as they were displayed on the same screen, interference also affected the same modality. This is an important difference between the proposed procedures and many of the flanker tasks previously used in studies involving emotional stimuli [40,43,44,47,48,62,63].

The above-described pattern of results supports the two-level model of interference control proposed in the introduction. On the plateau of congruency, the properties of the displayed stimuli are assessed, leading to the slowdown of response, when the properties of additional objects do not match the properties of the target object. Furthermore, when the content of the additional objects matches their disturbing properties, it may cause a significant decrease in the quality of performance in the task. On the plateau of emotional processing, the meaning of the word with its connotations is analysed. When it is loaded with an emotional charge (charge of arousal in particular), it slows down the deliverance of the response. The two plateaus do not overlap.

The study has its limitation. One of them refers particularly to the design. It is important to remember that the stimuli used in this study were divided into levels on dimensions of valence and arousal, which were crossed orthogonally. This process broke the naturally occurring relation between these two dimensions, which may be described as a U-shaped function, where negative and positive stimuli are charged with arousal [33]. Recreating this kind of relation in a set of targets used for an experiment could show a different shape of results. Although there are some limitations, the design allowed us to verify that the described effects are not dependent on the subjective significance of the certain word, its length or the frequency of usage. Another limitation is related to the sample choice. The study was run on students, which result in a highly homogenous and specific sample. However, sample diversity could contaminate the study results, as cognitive abilities deteriorate with age and are influenced by education. Therefore, narrowing the sample to students only allows minimalizing some confounding factors.

The study design was quite complex. The number of trials and hence, the length of the study can be treated as a kind of challenge in maintaining cognitive control. We might expect the impact fatigue on obtaining results, however this effect was minimalizing thanks to full randomization of trials and groups of words. Otherwise, the applied design allowed for controlling all three factors simultaneously. On the other hand, the study did not include overseeing of other possible confounding factors such as concreteness and abstractness, because it was not possible to include further factors in an already extensive scheme. It seems worth to consider this dimension in the further research plan, as some research proved higher concreteness is related with faster proceeding [67–70]. On the other hand, controlling this factor did not significantly affect the impact of other emotional stimuli properties such as valence and origin in EST, see e.g. Imbir and colleagues 2017 [71]. Additionally, in further research, we could replicate the applied procedure including other dimensions of emotionality like origin or dominance. Furthermore, the verbal material was built from nouns only, verifying if obtained results are replicable on adjectives or verbs could be an interesting further direction.

In conclusion we have to state, that the study revealed the effect of congruence, longer reaction times for incongruent trials occurred in both experiments. We also obtained an effect of arousal and the interaction effect of arousal and valence. The combination of two paradigms allowed on the operationalization of two different types of interference appearing on two different levels of processing. We believe that obtained results shed some new light in emotions role in cognitive processing.

## Supporting information

S1 Appendix (XLSX)
